# Seasonality of Serum Allergen-Specific IgE Levels in Scandinavian Dogs Suspected of Allergy

**DOI:** 10.3390/vetsci13060522

**Published:** 2026-05-28

**Authors:** Tilda Börjesson, Joe Streets, Thierry Olivry

**Affiliations:** 1 AniCura Falu Djursjukhus, Samuelsdalsägen 2B, 791 61 Falun, Sweden; tilda.borjesson@anicura.se; 2 Nextmune UK Labs, Unit 651, Street 5, Thorp Arch Trading Estate, Wetherby LS23 7FZ, UK; joestreets@gmail.com; 3 Nextmune AB, Riddargatan 17, 114 57 Stockholm, Sweden

**Keywords:** allergen, allergy, Denmark, IgE, mites, Norway, pollen, serological testing, Scandinavia, Sweden

## Abstract

Canine atopic dermatitis (AD) is a common allergic skin disease for which identifying allergen sensitization via IgE serological or intradermal testing is necessary to implement allergen-specific immunotherapy. Because serum IgE has a short half-life and circulating levels fluctuate with environmental allergen exposure, the timing of blood sampling may influence serological test outcomes. This cross-sectional study assessed seasonal variation in allergen-specific IgE concentrations and seropositivity rates across pollen and mite allergen categories in Scandinavian dogs suspected of allergic disease. PAX multiplex macroarray results from 5014 canine sera submitted by veterinarians across Denmark, Norway, and Sweden over one full year were retrospectively analyzed for 17 allergens, including tree, grass, and weed pollens, house dust mites, and storage mites. Mean sIgE levels showed statistically significant but relatively small seasonal variation. Seropositivity rates, however, showed clearer patterns: seropositivity for tree and weed pollen was highest in spring and summer, and that for house dust mite peaked in autumn and winter, while storage mite sensitization rates showed the opposite trend. These results confirm that IgE serological test results in dogs are affected by the sampling season and emphasize the importance of considering timing when collecting and interpreting IgE serological tests in atopic dogs.

## Simple Summary

Dogs with allergies often need immunotherapy injections tailored to the substances (allergens) they react to, and a serum test is often used to identify those triggers. However, IgE “allergic” antibodies do not remain elevated in the blood for long and fluctuate with allergen exposure. This means that the season when blood is drawn could affect test results. To investigate this, we analyzed more than 5000 IgE blood test results from dogs across Scandinavia over a full year. While mean IgE levels themselves changed only slightly between seasons, the number of dogs testing positive for specific allergens shifted more noticeably: pollen sensitization was most detectable in spring and summer, house dust mite reactivity peaked in autumn and winter, and storage mites showed the opposite trend. In short, the timing of sample collection is important, and these observations should be kept in mind when running and interpreting IgE blood tests.

## 1. Introduction

Canine atopic dermatitis (AD) has been re-defined recently by the International Committee of Allergic Diseases of Animals (ICADA) as “a hereditary, typically pruritic and predominantly T-cell driven inflammatory skin disease involving the interplay between skin barrier abnormalities, allergen sensitization, and microbial dysbiosis” [1]. Canine AD is often accompanied by primary and secondary lesions in a characteristic distribution [2], even though deviations from the typical phenotype frequently occur in some breeds [3,4]. To date, there is no reliable diagnostic test for canine AD, and the diagnosis is therefore made by combining historical information, the type and distribution of clinical signs, and by excluding other pruritic skin diseases that resemble it, especially cutaneous ectoparasitoses and microbial infections [2].

To prevent the recurrence of clinical signs associated with allergen sensitization, clinicians may select allergen-specific immunotherapy (AIT), for which identification of IgE sensitization to environmental allergens is required [5,6]. The determination of the aeroallergen sensitization repertoire in dogs with AD is usually performed using allergen-specific intradermal (IDT) tests and/or IgE serological tests (ST) [6,7].

For decades, IDTs and STs have used crude extracts, such as those from whole-house dust mites, fungal cultures, or entire pollen grains, which are difficult to standardize [8]. To reduce heterogeneity in the composition of crude extracts used to test humans [8] and animals with allergies [9], there has been increased interest in performing STs with native or recombinant molecular allergens [10,11]. The Pet Allergy Xplorer (PAX, Nextmune, Stockholm, Sweden) is a recently developed allergen-multiplex macroarray designed for allergy-suspected dogs, cats, and horses [12]. Like its predecessor for humans, the Allergy Xplorer (ALEX [12]—now in its third version [13]—Macroarray Diagnostics, Vienna, Austria), the PAX combines one-third allergen extracts and two-thirds molecular components to help characterize the IgE sensitization profile of animals suspected of allergy.

Serological tests are best performed when serum samples are expected to have high levels of specific IgE (sIgE) against the allergens that trigger allergy flares. Unfortunately, because IgE has a relatively short half-life in the serum [14], its circulating levels are expected to fluctuate with the patient’s exposure to environmental allergens, which can vary between seasons. This fluctuation was clearly demonstrated in two large-scale studies of human patients with allergies. In the first study, bimonthly evaluations of Timothy grass (*Phleum pratense*) sIgE serum concentrations revealed the highest yearly averages in May-June for patients from southern Europe and in July-August for those from northern European countries [15]; sIgE against silver birch (*Betula verrucosa*) allergens peaked in May-June compared with the rest of the year [15]. As house concentrations of mite group 1 allergens can increase in the summer [16], when mites proliferate during periods of high heat and humidity, mite-specific IgE levels in patients should logically rise in the ensuing season. This was clearly seen in one study in which the *Dermatophagoides pteronyssinus* IgE serum levels of over 24,000 allergy-suspected human patients showed a clear seasonal pattern, with an increase starting in September and peaking in November, and the lowest levels observed between February and August; the difference in sIgE levels between July and November was nearly two-fold [17].

We found only two small case studies documenting fluctuations in pollen-specific IgE serum concentrations in dogs across seasons. In the first paper reporting the use of ST in atopic dogs, ragweed-specific IgE levels peaked near the end of the ragweed pollination season (September); in another dog, however, they remained stable throughout the year [18]. In the second article, sIgE levels to the major allergen Cry j 1 in three dogs with seasonal allergies to Japanese cedar pollen were higher during the tree’s pollination season (March) than in the autumn [19].

Results from other studies suggest that, presumably due to fluctuations in allergen-specific IgE serum levels, sensitization positivity rates may vary between seasons in dogs suspected of having allergies. For example, in a cross-sectional Norwegian survey of atopic dogs, serum samples collected in the summer and autumn showed higher test positivity rates than those gathered in the spring and winter; this pattern was observed for both indoor (i.e., mite) and outdoor (i.e., pollen) allergens [20]. In Thailand, sensitization rates of atopic dogs to indoor allergens (mites and *Malassezia* yeast)—but not pollen extracts—were higher during the rainy season than during winter [21]. Finally, in a large survey of more than 25,000 serological tests on French dogs suspected of having AD, significantly higher proportions of positive STs (for all allergens) were observed in spring and summer than in winter [22]. When results were separated into broad allergen categories, significantly higher positivity rates were observed for pollen extracts in spring, summer, and autumn, and for mite extracts in summer, compared with winter [22].

In contrast to the reports above, which showed seasonal variations in positivity frequencies for some (or all) allergen categories, an older study found no changes in monthly positivity rates for any of the 10 pollen allergen groups tested [23]. Similar conclusions were reached more recently: a study of 211 dogs with AD found no statistical differences in positivity rates across allergen groups between sampling seasons, regardless of whether IDT or ST was used [24]. A lack of variability in IDT positivity rates to grass pollen between seasons has been reported previously in France [25].

The recent introduction of the PAX, a quantitative ST that uses molecular allergen components and a unique method to measure the neutralizing effectiveness of IgE against cross-reactive carbohydrate determinants (CCDs), offers an opportunity to reevaluate seasonal variations in IgE levels and seropositivity rates.

This cross-sectional study, therefore, aimed to evaluate seasonal variability in the rates of allergen-specific IgE and PAX seropositivity for several pollen and mite allergens in the serum of Scandinavian dogs suspected of allergic disease. This report, to the authors’ knowledge, represents the first comprehensive study of the seasonal impact on allergen-specific IgE results using some molecular allergens in dogs.

## 2. Materials and Methods

### 2.1. PAX Test Results

We compiled PAX test results from canine sera submitted by veterinarians across Scandinavia (Denmark, Norway, and Sweden) between 1 March 2023, and 28 February 2024. Among the 247 individual allergen spots available in that version of the PAX for dogs (22.2) (see Table S1 in [26] for the list of allergens), we selected representative allergen extracts or components in the categories of interest (Table 1). These allergens were chosen because they commonly sensitize dogs in Europe (Nextmune, unpublished data) and, for pollens, the originating plants have been observed in at least one of the three Scandinavian countries since the beginning of this century.

Because plant CCDs can sensitize dogs and lead to the production of false-positive, potentially clinically irrelevant, pollen-specific IgE in dogs [27], we first removed all canine sera with serum IgE levels of at least 28 ng/mL to one or both “CCD-IgE detectors”, which remained after the application of the PAX’s standard CCD-IgE blocking strategy [26]. Altogether, 5014 PAX test results remained available for analysis.

### 2.2. Parameters and Grouping

For each allergen listed in Table 1, we extracted two parameters from all PAX results. The first was the IgE level in ng/mL, which was then averaged across dogs. The second was the calculation of the sensitization positivity rate (i.e., seropositivity rate) for all sera using the standard PAX threshold of 28 ng/mL [26].

The two parameters above were compared between the seasons deemed most relevant to the pollination of the chosen plants in Scandinavia [28]:

- Spring: March, April, and May 2023;
- Summer: June, July, and August 2023;
- Autumn: September, October, and November 2023;
- Winter: December 2023, January, and February 2024.

### 2.3. Statistical Analyses

IgE levels for each allergen, whether analyzed individually or grouped by category (first column in Table 1), were initially analyzed across seasons using ANOVA, followed by a post hoc Tukey test. Seasonal seropositivity percentages for each allergen and allergen category were also examined; for the latter, we considered a category positive if at least one allergen within that category exceeded the threshold. After binarizing the data (positive or negative), a Chi-squared test was performed. Statistical significance was defined as a *p*-value of 0.05 or lower. All statistical analyses were performed in the R programming language using base R functions from the stats package. Data handling, validation, and figure generation were performed using the dplyr, tidyverse, and ggplot2 packages.

## 3. Results

### 3.1. Allergen-Specific IgE Levels

**Figure 1:**
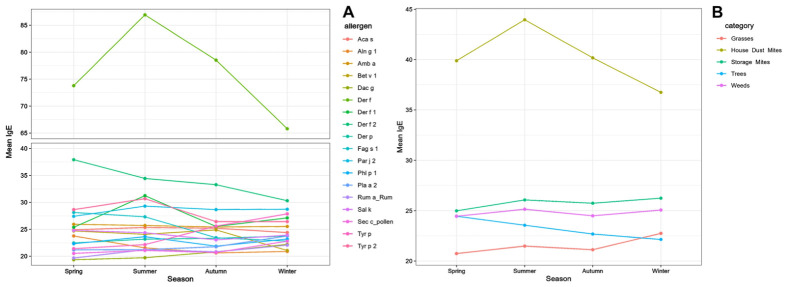
Mean allergen-specific IgE levels by season: (**A**) for individual allergens; (**B**) for allergen categories.

The mean sIgE levels for individual allergens and different allergen categories are shown in Figure 1A and Figure 1B, respectively, with more details being provided in Table S1.

Comparisons of sIgE levels, whether analyzed individually or grouped by category, are presented in the various elements of Table S2. The leftmost panel displays the ANOVA results, which identified significant differences in sIgE values for grass pollen and house dust mite allergen categories across seasons. For specific allergens, notable seasonal differences in sIgE levels were observed for the tree pollen allergens Aln g 1, Fag s 1, and Pla a 2; for the grass allergens Dac g and Sec c; and for pollen from the weeds Rumex and Sal k. Additionally, significant seasonal differences were found for Der f house dust mites (HDMs) and allergens from Tyr p storage mites (SMs).

The two-by-two statistical comparisons of allergen-specific IgE levels across seasons, performed using Tukey post hoc tests, are shown in the middle (individual allergens) and rightmost panel (allergen categories) of Table S2. In summary, sIgE levels for two of four individual tree pollen allergens (Aln g 1 and Fag s 1, both related PR-10 family allergens) were significantly higher in spring and summer, whereas those of the unrelated Pla a 2 allergen were significantly higher in the autumn and winter than in the other two seasons.

Specific IgE levels against two grass extracts and the grass category were elevated in winter compared with the other seasons. Specific IgE against Sal k was notably lower in winter than in the other three seasons. Significantly higher Der f-specific IgE levels were observed in the summer than in the winter. The comparison between IgE levels for Tyr p extract and Tyr p 2 yielded conflicting results: Tyr p-specific IgE levels were higher in autumn and winter than in spring and summer, whereas those against Tyr p 2 were significantly higher in summer than in autumn and winter.

Although the mean sIgE values and the extent of seasonal variation in sIgE levels appear modest, this is because most tested sera had low sIgE levels against these allergens, suggesting that most dogs were not sensitized to individually tested allergens.

### 3.2. Seropositivity Rates

**Figure 2:**
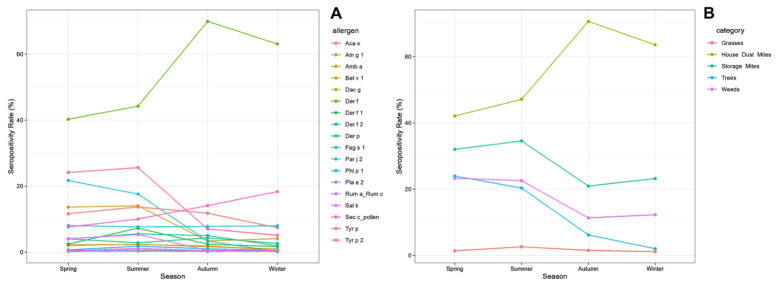
Seropositivity rates by season: (**A**) for individual allergens; (**B**) for allergen categories.

Using the standard PAX positivity threshold of 28 ng/mL, the seropositivity rates for individual allergens and their categories across seasons are presented in Figure 2A,B and Table S3.

Overall, statistically significant differences were observed in IgE seropositivity rates across all allergen categories and individual allergens, except for Dac g, Sec c, Rumex pollen extracts, and the pollen components Pla a 2 and Par j 2 (Table S4; leftmost table).

The seropositivity rates by allergen category showed statistically significant differences across seasons when compared two-by-two (see Table S4, center panel). Specifically, tree and weed pollen allergen positivity rates were notably higher in spring and summer than in autumn and winter. In contrast, there were no significant seasonal differences in grass pollen allergen positivity rates. Sensitization to HDMs was significantly more common in autumn and winter than in spring and summer, whereas that to SM allergens showed the opposite pattern.

The statistical comparison of PAX positivity rates for individual allergens across seasons, two-by-two, showed significant differences that mirrored those observed for allergen categories (Table S4, rightmost panel). These seasonal differences in sensitization rates across allergen categories were primarily driven by one or more dominant allergens within each category. For example, higher positivity rates to tree pollen allergens in the spring and summer were mainly due to variations in sensitization to Fag s 1, with smaller contributions from Aln g 1 and Bet v 1. For weed pollens, the differences were mainly due to the Amb a and Sal k extracts. Likewise, higher positivity rates for HDM allergens in autumn and winter were strongly associated with those to the Der f extract. For SMs, higher sensitization detection in the spring and summer was mostly attributed to Aca s and Tyr p 2, although the positivity rates for the Tyr p extract varied in the opposite pattern (Table S3).

## 4. Discussion

In this cross-sectional study, we investigated the seasonal variations in sIgE against selected pollen and mite allergens in a cohort of Scandinavian dogs. The analysis of mean sIgE concentrations revealed significant fluctuations within broad categories and for several individual components. A more detailed analysis showed that sIgE titers for two tree pollen allergens, Aln g 1 and Fag s 1, peaked during the spring, aligning with regional pollination phenology and established data from human populations [15,22,28,29]. Conversely, sIgE titers to the grass allergens Dac g and Sec c increased unexpectedly during the winter months. Although these observations may seem counterintuitive and differ from expected pollination patterns and previous seasonal reports, the extent of these variations was modest, even though they were statistically significant [15,22,28,29]. It is unclear whether these subtle changes in sIgE levels will be clinically relevant, especially since the severity of clinical signs in human AD is not correlated with allergen-specific IgE or total IgE levels [30].

Nevertheless, a small difference in seasonal sIgE levels is consistent with results from previous research. For instance, while a large human cohort exhibited elevated sIgE levels against grass pollen during the spring and summer, the increase was only small compared to winter values [15]. A somewhat counterintuitive observation in our analysis of the present results was that some dogs exhibited high sIgE levels to grass allergens even when active seasonal pollination was absent. These observations suggest that dogs may be exposed to pollen allergens well beyond the peak pollination period, perhaps due to contact with pollen on the ground, a hypothesis supported by Fraser et al., who identified a variety of pollen grains in canine fecal samples even during winter months, pollen that might have been licked on the pets’ coats and then ingested [31].

In addition to those against pollen allergens, our analyses revealed significant seasonal fluctuations in sIgE levels for two extracts from different mite species (Der f and Tyr p) and for the specific allergen component Tyr p 2. Differences in observed parameters between an extract and a component for the same organism are likely due to the extract containing thousands of proteins and, at times, low quantities of important allergenic components [9]. The discrepancy between the seasonal changes in Tyr p and Tyr p 2 is likely due to the Tyr p extract cross-reacting with that of Der f [32], while Tyr p 2 does not do so with Der f 2 [33]. Nevertheless, these findings are consistent with previous research in human medicine documenting seasonal variations in sIgE levels directed against HDMs [16,17]. Here again, despite statistically differing between seasons, the mean sIgE levels for mite allergens remained relatively stable over time, mirroring the trend observed for pollen allergens. These small variations in IgE levels, which we were able to detect as significant given the large number of dogs tested, might still reflect a seasonal exposure to HDMs, which are likely to proliferate and release more allergens during the warmer months, leading to higher sIgE levels in the summer.

The comparison of seropositivity rates across seasons also revealed significant fluctuations across all allergen categories and for most individual allergens. These differences were more pronounced than those observed in mean sIgE concentrations. Using the standard PAX threshold (28 ng/mL), we demonstrated that the prevalence of positive samples in trees and weeds peaked in spring and spring/summer, respectively, during the plants’ expected pollination seasons. The significant contributions of Aln g 1, Bet v 1, Fag s 1, Amb a, and Sal k suggest that these allergens exhibit distinct seasonal cycles. Notably, while the broad grass category remained relatively stable throughout the year, the elevated sensitization rates to Phl p 1 during the summer underscore the clinical utility of molecular diagnostics in identifying seasonal shifts that might otherwise be obscured at the extract level. Our findings are consistent with two previous studies that identified seasonal fluctuations in pollen-specific seropositivity rates in Europe [20,22]. Conversely, Canning et al. reported no significant seasonal influence on IgE seropositivity rates in the US [24]. The observed differences may be explained by substantial variation in population sizes, as well as by differences in analytical methods, range of selected allergens, geography, and climate.

In the current population of Scandinavian dogs, the sensitization rate to HDMs was higher in autumn and winter than in spring and summer, whereas the sensitization rate to SMs, surprisingly, exhibited the opposite seasonal pattern. Interestingly, this trend contrasts with the results obtained when using mean sIgE concentrations. The reasons for this parameter discrepancy across mite categories are unknown, but they could be related to differential exposure to mite allergens, as, for example, HDM allergens have recently been found not only indoors but also in outdoor aerosols [34]. For pollen allergens, seasonal fluctuations were more pronounced in seropositivity rates than in mean sIgE values. While several studies have documented a seasonality in mite sensitization detection rates, most of these reports aggregated allergens into broad categories (e.g., “mites” or “pollen”) rather than reporting data for individual species or molecular components, which complicates direct comparisons with our results [20,21,22,24].

Nevertheless, our observation of an autumn and winter peak in HDM seropositivity contrasts with results reported by Drouet et al. and Bjelland et al., who detected higher HDM seropositivity rates in spring and summer. A summer peak in HDM allergen exposure is biologically plausible, as mites require high humidity and elevated temperatures for optimal proliferation [16,35]. However, previous research identified a two-month latency between peak environmental humidity and subsequent increases in mite populations, which may explain why the rise in allergen-specific IgE against HDM in the present study occurred after the initial seasonal climate shift. Furthermore, IgE sensitization is influenced by a multitude of factors beyond seasonal variation, including UV radiation, geographical location, and lifestyle-related variables, but these parameters were not assessed in our study [17]. Nevertheless, we speculate that increased time spent indoors during the autumn and winter months might intensify the exposure to HDM fecal allergens, which may then contribute to the observed rise in seropositivity during the colder seasons. Additional reasons for discrepancies between our results and those of other studies include a variability in testing methodology (e.g., different allergen extracts, test sensitivity, positivity thresholds) and differences in geography or climate.

While the present study provides a deeper understanding of the seasonal fluctuations of allergen-specific IgE in dogs, several limitations inherent to the study design must be acknowledged. Firstly, we did not have access to clinical information for the dogs whose serum samples were analyzed in this study, so we did not include it. These dogs probably showed a variety of clinical phenotypes, from AD to food allergy with only gastrointestinal signs; some might have even been incorrectly suspected of being allergic. However, most dogs likely exhibited signs indicative of allergy, as the PAX is typically performed to detect sensitizations before starting immunotherapy. Due to the uncertainty surrounding their definitive diagnoses, we elected to refer to the study subjects as “allergy-suspected dogs” in this paper. Secondly, some of the dogs tested may have been treated with anti-allergic drugs, which are largely immunosuppressive in their mechanism of action, and these interventions could potentially have negatively affected sIgE levels. Thirdly, the use of molecular technology and advanced CCD blocking likely prevents direct comparison with previous studies that employed different analytical methods. One should also note that the timing of sampling likely reflects the onset of clinical symptoms rather than the precise moment of peak environmental allergen exposure, which therefore may influence the interpretation of the results. Finally, because sensitizations to environmental allergens are biologically interrelated, the statistical tests were not entirely independent. Nevertheless, the large number of comparisons in this study may have increased the likelihood of chance findings; therefore, any individual statistically significant association should be interpreted with caution. To address these limitations, future longitudinal studies that repeatedly test the same individuals over an annual cycle would be highly valuable.

## 5. Conclusions

This study provides novel insights into the seasonal variations in canine sIgE levels, with significant differences observed in mean sIgE levels and seropositivity rates across seasons for both pollen and mite allergens. While mean sIgE levels varied significantly, the magnitude of these variations was subtle and may not be clinically relevant. In contrast, comparisons of seropositivity rates revealed pronounced fluctuations across all broad categories and for the majority of individual allergens. Consequently, our findings suggest that the timing of serological testing should be considered during the diagnostic evaluation of dogs with suspected allergic disease.

## Figures and Tables

**Table 1:** Allergens selected for analysis.

	Allergen Name	Organism (Latin)	Organism (English)	Extract (E) or Component (C)
Tree pollens	Aln g 1	*Alnus glutinosa*	Alder	C
	Bet v 1	*Betula verrucosa*	Birch	C
	Fag s 1	*Fagus sylvatica*	Beech	C
	Pla a 2	*Platanus acerifolia*	Sycamore/plane tree	C
Grass pollens	Dag g	*Dactylis glomerata*	Cocksfoot/orchard grass	E
	Phl p 1	*Phleum pratense*	Timothy grass	C
	Sec c_pollen	*Secale cereale*	Rye grass	E
Weed pollens	Amb a	*Ambrosia artemisiifolia*	Ragweed	E
	Par j 2	*Parietaria Judaica*	Wall pellitory	C
	Rum a_Rum c	*Rumex acetosella* + *Rumex crispus*	Yellow dock + sorrel	E mix
	Sal k	*Salsola kali*	Russian thistle	E
House dust mites	Der f	*Dermatophagoides farinae*	American house dust mite (HDM)	E
	Der p	*Dermatophagoides* *pteronyssinus*	European HDM	E
	Der f 1	*Dermatophagoides farinae*	American house dust mite (HDM)	C
	Der f 2	*Dermatophagoides farinae*	American house dust mite (HDM)	C
Storage mites	Aca s	*Acarus siro*	-	E
	Tyr p	*Tyrophagus putrescentiae*	-	E
	Tyr p 2	*Tyrophagus putrescentiae*	-	C

## Data Availability

The original contributions presented in this study are included in the article/Supplementary Materials. Further inquiries can be directed to the corresponding author.

## Supplementary Materials

The following supporting information can be downloaded at: https://www.mdpi.com/article/10.3390/vetsci13060522/s1. Table S1: Seasonal variation in specific IgE serum levels per individual allergen and allergen category; Table S2: Statistical comparison of specific IgE serum levels between seasons: ANOVAs and post hoc Tukey tests; Table S3: Seasonal variation in seropositivity rates per individual allergen and allergen category; Table S4: Statistical comparison of seropositivity rates between seasons: Chi-square tests.

## Abbreviations

The following abbreviations are used in this manuscript:

AD	Atopic dermatitis
AIT	Allergen (specific) immunotherapy
ALEX	Allergy Xplorer
CCDs	cross-reactive carbohydrate determinants
ELISA	Enzyme-linked immunosorbent assay
HDM	House dust mite
IDT	Intradermal testing
ICADA	International Committee of Allergic Diseases of Animals
IgE	Immunoglobulin E
PAX	Pet Allergy Xplorer
SM	Storage mite
ST	Serological test
